# Supplementary material for: Arboviruses and symbiotic viruses cooperatively hijack insect sperm-specific proteins for paternal transmission
Source: Nat Commun. 2023 Mar 9;14:1289. doi: 10.1038/s41467-023-36993-0 (PMC9998617; doi:10.1038/s41467-023-36993-0)

SUPPLEMENTARY INFORMATION

Arboviruses and symbiotic viruses cooperatively hijack insect sperm-specific proteins for paternal transmission

Wan et al.

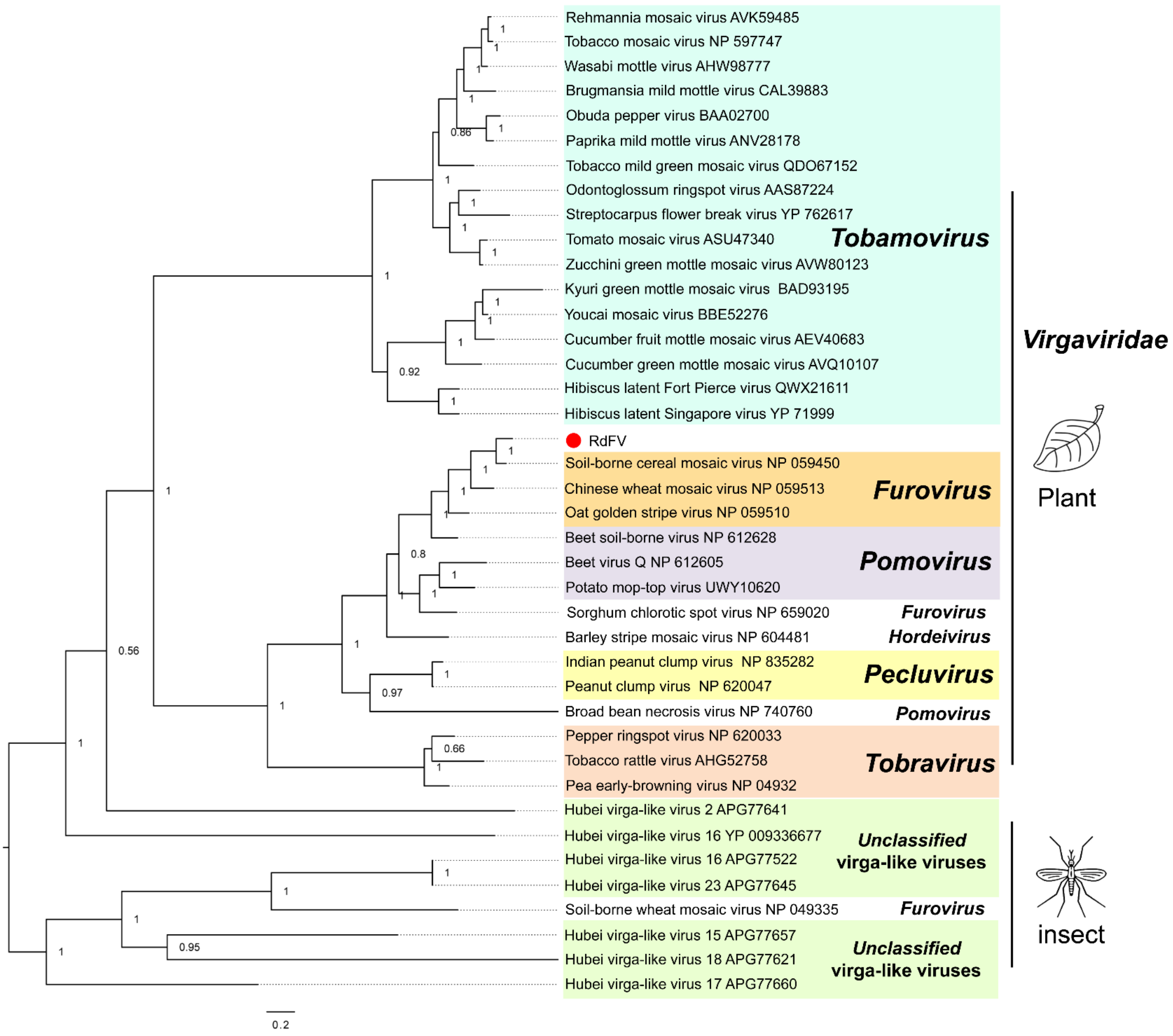

Fig. S1 Phylogenetic relationships of RdFV with counterparts in *Virgaviridae* family and insect-specific virga-like viruses. The available amino acid sequences of RdFV RdRp and counterparts were analyzed for reconstruction of Mid-point rooted Bayesian phylogenetic trees. Bayesian posterior probabilities are given above each node. The bar represents numbers of substitutions per site.

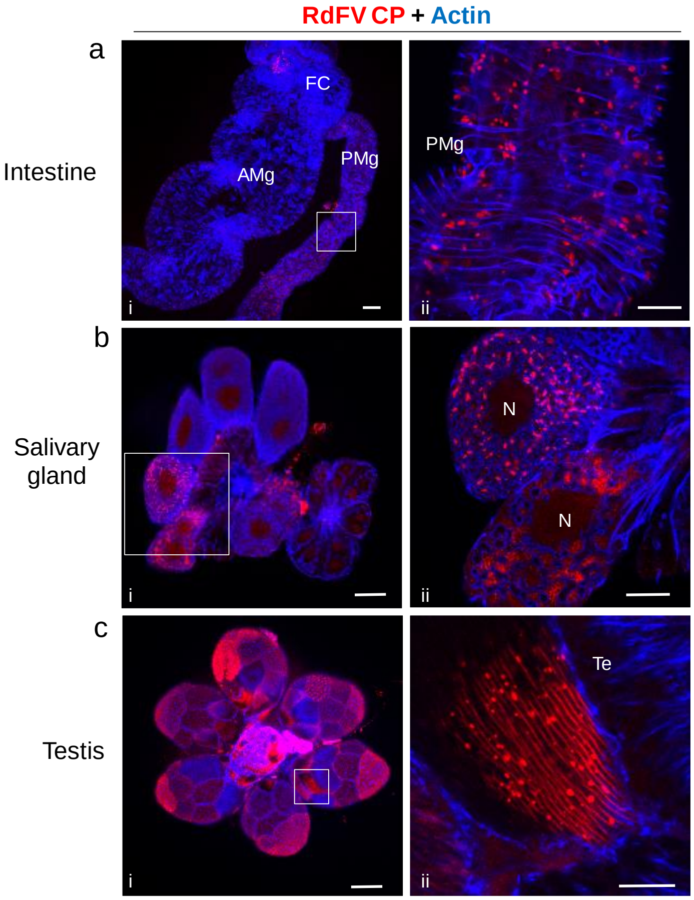

Fig. S2 Immunofluorescence microscopy showing RdFV infection in various organs of *R. dorsalis*. Intestines, salivary glands or testes of RdFV-positive leafhoppers were dissected, immunostained with CP-rhodamine (red) and actin dye phalloidin-Alexa Fluor 647 carboxylic acid (blue), and then processed for immunofluorescence microscopy. Panels ii are the enlargement of the boxed areas in panels i, respectively. FC, filter chambler. AMg, anterior midgut. PMg, posterior midgut. Te, testis. N, nucleus. Bars, 10 μm.

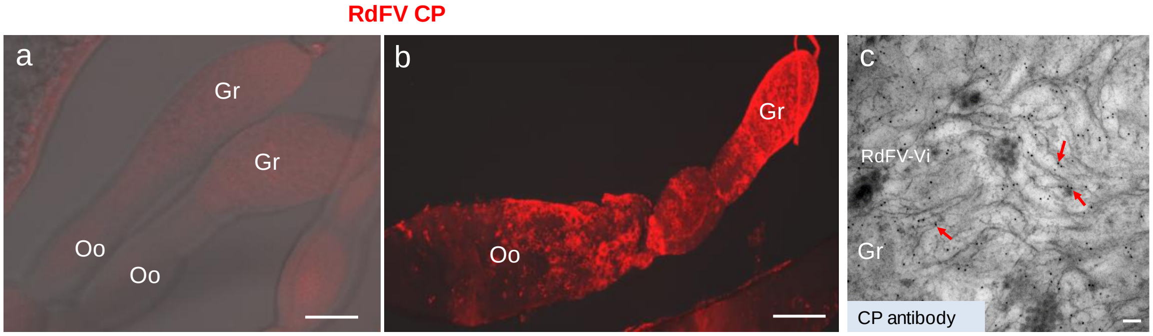

Fig. S3 RdFV infection in ovaries of *R. dorsalis*. a, **b** Immunofluorescence microscopy showing the distribution of RdFV in female ovary. The virus-free ovary served as a control (**a**). Female ovaries were immunostained with CP-rhodamine (red), and then processed for immunofluorescence microscopy. Oo, oocyte. Gr, germarium. Bars, 70 μm. **c** Immunoelectron microscopy showing the presence of RdFV in female ovary. Female ovaries were immunolabeled with CP-specific IgG as the primary antibody, followed by treatment with 15-nm gold particle-conjugated IgG as the secondary antibody. Red arrows indicate gold particles. Gr, germarium. Vi, virions. Bar, 100 nm.

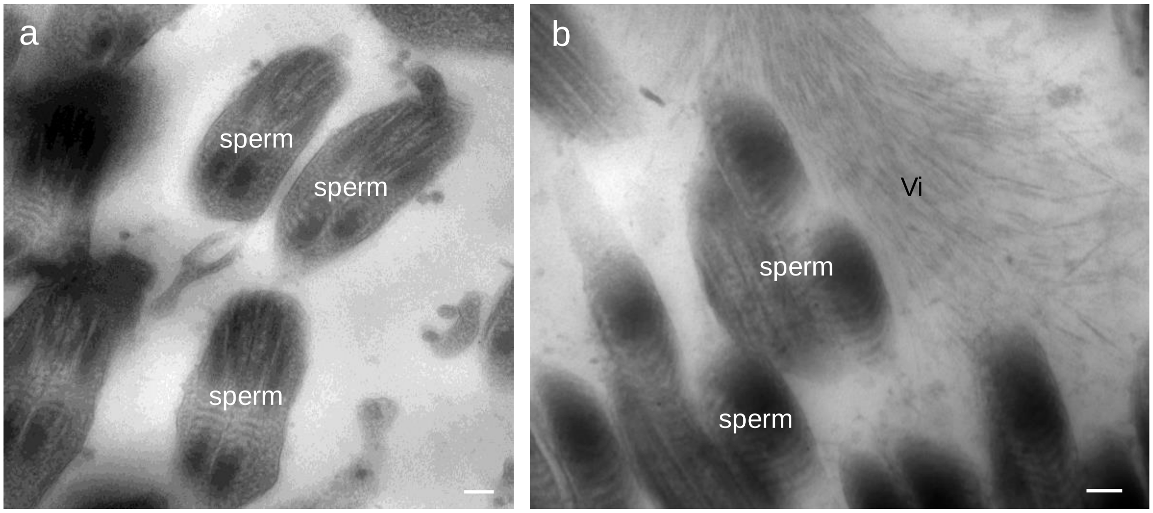

Fig. S4 Effect of RdFV on sperm morphology as determined by electron microscopy. Testes of RdFV-free (**a**) or positive (**b**) males were excised and processed for electron microscopy. Vi, virions. Bars, 100 nm.

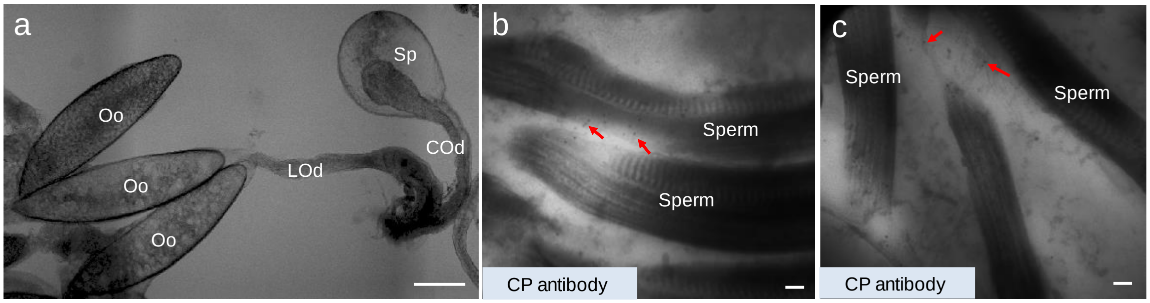

Fig. S5 RdFV infection in spermatheca of female *R. dorsalis* at 5-day post mating with RdFV-positive males. a Transmitted light micrograph of reproductive organs of RdFV-free females, including spermatheca (Sp), common oviduct (COd), lateral oviduct (LOd) and oocyte (Oo). Bars, 200 nm. **b**, **c** Immunoelectron microscopy showing the presence of virus-associated sperms in female spermatheca. Spermathecas of females at 5-day post mating with RdFV-positive males were immunolabeled with CP-specific IgG as the primary antibody, followed by treatment with 15-nm gold particle-conjugated IgG as the secondary antibody. Red arrows indicate gold particles. Bars, 100 nm.

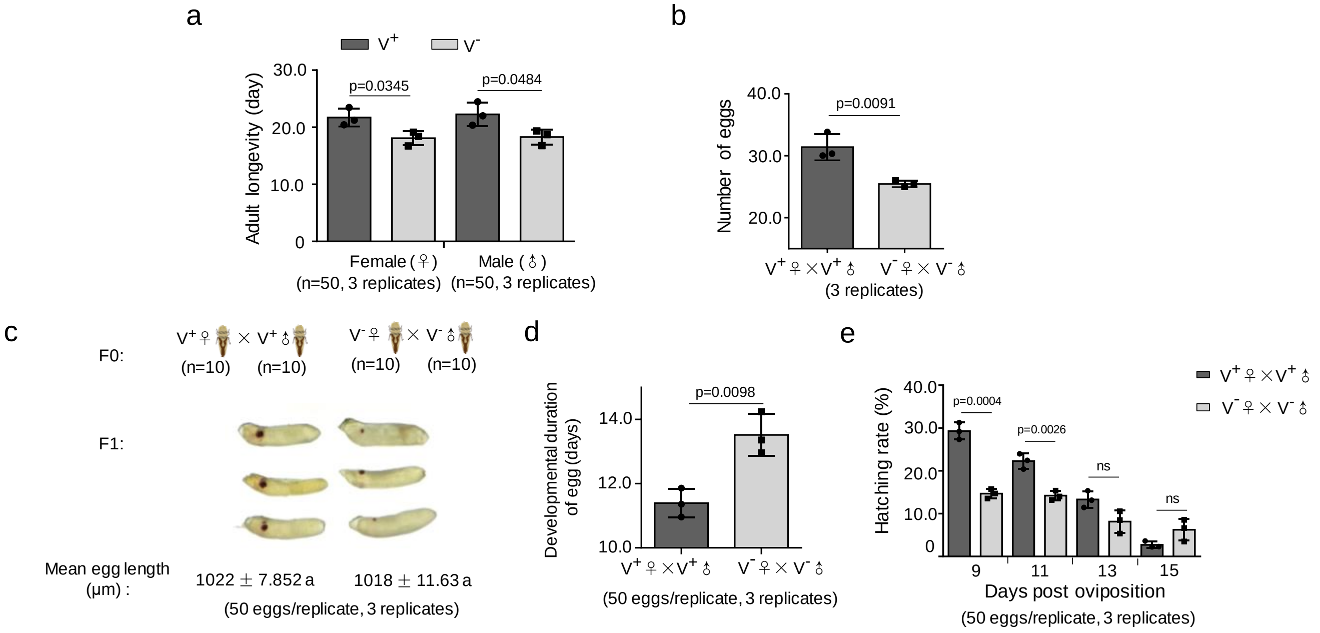

Fig. S6 Effects of vertical transmission of RdFV on fitness of *R. dorsalis* adults and offspring. Mating combinations were established as follows: infected virgin female × infected male, and uninfected virgin female × uninfected male. **a** Effects of RdFV infection on the longevity of male and female adults. Longevity of 50 RdFV-free or positive female or male adults was analyzed. Means (± SD) are shown from 50 insects, and represent three replicates (two-tailed t-test). **b**-**d** Progeny egg number (**b**), size (**c**), development duration (**d**) and hatching rate (**e**) of female adults from different mating combinations. Fifty eggs with red eyespots were randomly collected from each combination was measured for the length of each egg. Data are presented as mean (± SD) for three independent experiments of two mating combinations (two-tailed t-test). Ns, not significant.

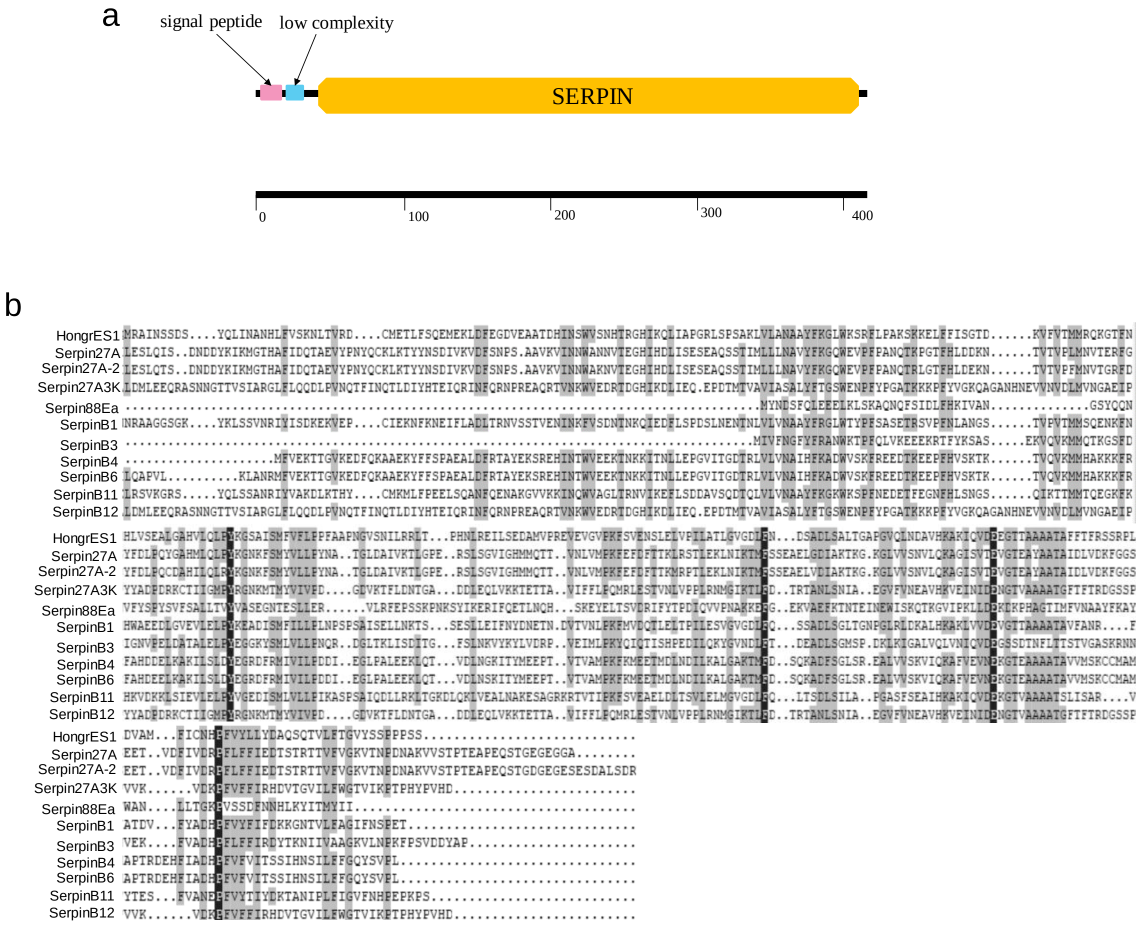

Fig. S7 Sequence analyses of HongrES1 amino acid. a Diagram of the domains of HongrES1 amino acid sequence as determined by the SMART analysis (http://smart.embl-heidelberg.de/). **b** Alignment of the conserved sequences of HongrES1 with serpin homologs of *R. dorsalis* analyzed by the DNAman 8.0 software. All conserved residues are highlighted in black.

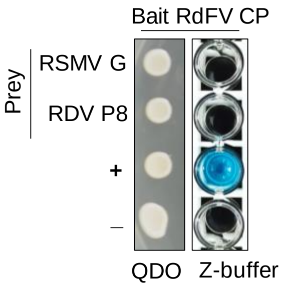

Fig. S8 Y2H assay showing the failure for interaction of RdFV CP with RSMV G or RDV P8. Transformants on QDO plates are labeled as follows: RSMV G, pPR3-N-RSMV G / pDHB1-CP; P8, pPR3-N-P8 (RDV) / pDHB1-CP; +, positive control, i.e., pLargeT / p53; –, negative control, i.e., pDHB1 / pRR3N. β-galactosidase assay was detected in Z buffer with X-Gal. QDO, SD/-Trp-Leu-His-Ade medium.

Supplementary Table 1 List of oligonucleotide primers for qPCR assays used in this study.

Oligonucleotide	Sequence (5'-3')	Tm (°C)
RD_EF1_F	CAGTGAGAGCCGTTTTGAG	58
RD_EF1_R	AGGGCATCTTGTCAGAGGGC	58
RGDV_P8_F	TGACCTTCATCGTCTCTGAGTCCGA	61
RGDV_P8_R	CGTTACCATTAACCGCGTTCACCTG	61
RdFV_CP_F	ATACGCAGTTCCGTCTCTTCCAAAC	60
RdFV_CP_R	CAGCCATGCCTTCAATATCTCATCA	60
HongrES1_F	ACTTTGCCCTGTCCATGCTCCAGAG	62
HongrES1_R	AAGTACGCCAGCAGCAGAGTGCTGT	62
OsActin_F	CAGCCACACCGTGCCAATCTATGAA	60
OsActin_R	TCAAGCTGTCAGTGAGGTCACGCCC	60

Supplementary Table 2 List of oligonucleotide primers except qPCR assays used in this study.

Oligonucleotide	Assay	Sequence (5'-3')
pDHB1_RGDV_P8_F	Y2H	ACGGCCAGGCCTCCATGGATGTCGCGCCAAGCTTGGATCGAGA
pDHB1_RGDV_P8_R	Y2H	GTTGATCTGGAGGGATCCTTAGTTTACTGTGTAATACCTACCG
pDHB1_RdFV_CP _F	Y2H	ACGGCCAGGCCTCCATGGATGTCATCACGTTCTCCTGCTCTGC
pDHB1_RdFV_CP _R	Y2H	GTTGATCTGGAGGGATCCTTACTGTTTACCACTCCCGGTACGT
pPR3-N_HongrES1_F	Y2H	GAGTGGCCATTACGGCCCGGGAAATGTTGGTGTTGCTAGTGTGTGTGG
pPR3-N_HongrES1_R	Y2H	GATAAGCTTGATATCGAATTCTTACGAGCTGGGGGGAGGGGAACTG
pPR3-N_ RdFV_CP_F	Y2H	GAGTGGCCATTACGGCCCGGGAAATGTCATCACGTTCTCCTGCTCTGC
pPR3-N_ RdFV_CP_R	Y2H	GATAAGCTTGATATCGAATTCTTACTGTTTACCACTCCCGGTACGT
pPR3-N_RSMV_G_F	Y2H	GAGTGGCCATTACGGCCCGGGAAATGATGAGGATTTCGGTCTTTCTCT
pPR3-N_RSMV_G_R	Y2H	GATAAGCTTGATATCGAATTCTTAGTCACTTTCTATAGTATATTCG
pPR3-N_RDV_P8 _F	Y2H	GAGTGGCCATTACGGCCCGGGAAATGTCACGCCAGATGTGGTTAGACA
pPR3-N_RDV_P8 _R	Y2H	GATAAGCTTGATATCGAATTCCTAATT

		TGGTCGATAGTATCTTCCA
pGEX-4T-3-F_ RdFV_CP_F	Pull-down	CGCGTGGATCCCCGAATTCCATGTCATCACGTTCTCCTGCTCTGC
pGEX-4T-3-F_ RdFV_CP_R	Pull-down	ACGATGCGGCCGCTCGAGTCTTACTGTTTACCACTCCCGGTACGT
pGEX-4T-3-F_HongrES1_F	Pull-down	CGCGTGGATCCCCGAATTCCATGTTGGTGTTGCTAGTGTGTGTGG
pGEX-4T-3-F_HongrES1_R	Pull-down	ACGATGCGGCCGCTCGAGTCTTACGAGCTGGGGGGAGGGGAACTG
pGEX-4T-3-F_ RGDV_P8_F	Pull-down	CGCGTGGATCCCCGAATTCCATGTCGCGCCAAGCTTGGATCGAGA
pGEX-4T-3-F_ RGDV_P8_R	Pull-down	ACGATGCGGCCGCTCGAGTCTTAGTTTACTGTGTAATACCTACCG
pEASY-Blunt E1_ HongrES1_F	Pull-down	ATGTTGGTGTTGCTAGTGTGTGTGG
pEASY-Blunt E1_ HongrES1_R	Pull-down	TTACGAGCTGGGGGGAGGGGAACTG
pEASY-Blunt E1_ RdFV_CP_F	Pull-down/ virus detection	ATGTCATCACGTTCTCCTGCTCTGC
pEASY-Blunt E1_ RdFV_CP_R	Pull-down/ virus detection	TTACTGTTTACCACTCCCGGTACGT
pEASY-Blunt E1_ RGDV_P8_F	Pull-down/ virus detection	ATGTCGCGCCAAGCTTGGATCGAGA
pEASY-Blunt E1_ RGDV_P8_R	Pull-down/ virus detection	TTAGTTTACTGTGTAATACCTACCG
dsHongrES1_F	RNAi	ATTCTCTAGAAGCTTAATACGACTCA

		CTATAGGGTGGACTTTGAGGGAGAC GTGGAGGC[a]
dsHongrES1_R	RNAi	ATTCTCTAGAAGCTTAATACGACTCA CTATAGGGGAGCAGGTAGACGAAGG GATGGTTG
dsRdFV_CP_F	RNAi	ATTCTCTAGAAGCTTAATACGACTCA CTATAGGGCGGGACCGTTATCTTCAA ACTTATC
dsRdFV_CP_R	RNAi	ATTCTCTAGAAGCTTAATACGACTCA CTATAGGGATCGGAAATCTGGGTATT TTTAGGC
dsGFP_F	RNAi	ATTCTCTAGAAGCTTAATACGACTCA CTATAGGGAAGTTCAGCGTGTCCGG CGA
dsGFP_R	RNAi	ATTCTCTAGAAGCTTAATACGACTCA CTATAGGGGAAGTTCACCTTGATGCC GTT

[a] Sequences with underlines indicate the T7 promoter.

Supplementary Table 3 Mating combination of females and males to test the paternal virus transmission.

Purpose	♀ ×	♂
RdFV vertical transmission	RdFV^{-} [a] or RdFV^{+} [b]	RdFV^{-} or RdFV^{+}
Knockdown of HongrES1 expression on paternal RdFV transmission	RdFV^{-}	dsGFP- or dsHongrES1-treated RdFV^{+}
Knockdown of HongrES1 expression on paternal RGDV transmission	RGDV^{-}/RdFV^{+}	dsGFP- or dsHongrES1-treated RdFV^{+}/ RGDV^{+}
Knockdown of RdFV CP expression on paternal RGDV transmission o	RdFV^{-}/RGDV^{-}	dsGFP- or dsCP-treated RdFV^{+}/ RGDV^{+}
Knockdown of RGDV P8 expression on paternal RdFV transmission	RdFV^{-}/RGDV^{-}	dsGFP- or dsP8-treated RdFV^{+}/ RGDV^{+}
Effect of RdFV on paternal RGDV transmission	RdFV^{-}/RGDV^{-}	RdFV^{-}/RGDV^{+} or RdFV^{+}/ RGDV^{+}
Effect of RdFV on insect and offspring fitness	RdFV^{-}/RdFV^{+}	RdFV^{+}/RdFV^{-}

[a] -, virus-free.

[b] +, virus-positive.

Uncropped blots used in the main figures

Fig. 1c

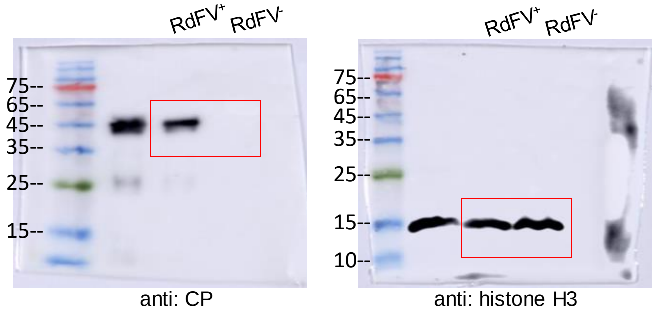

Fig.2b

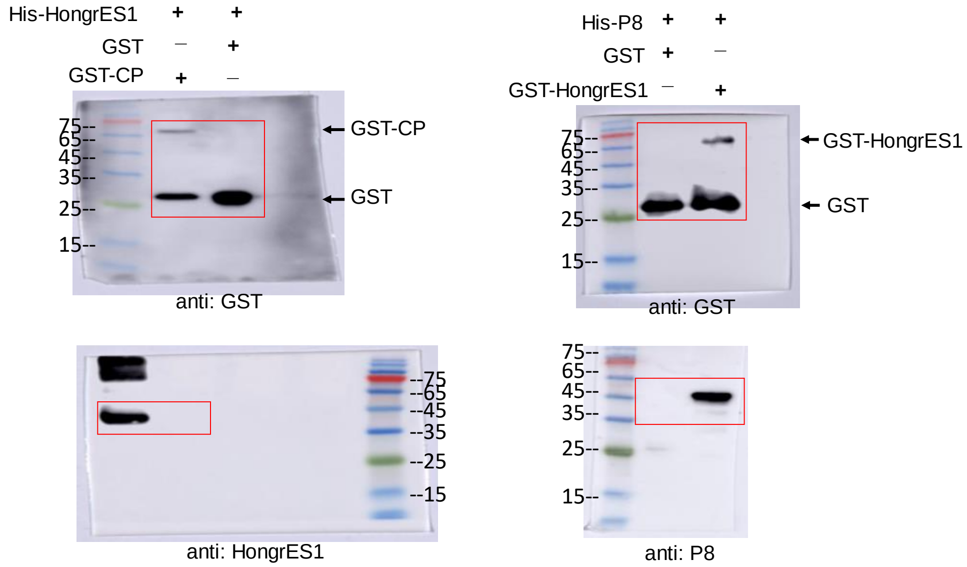

Fig.2d

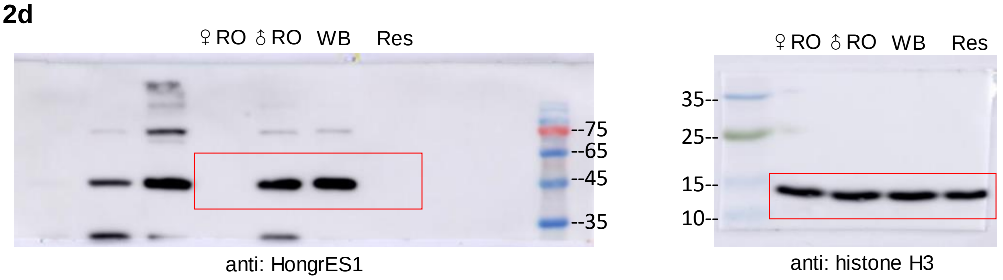

Fig. 2g

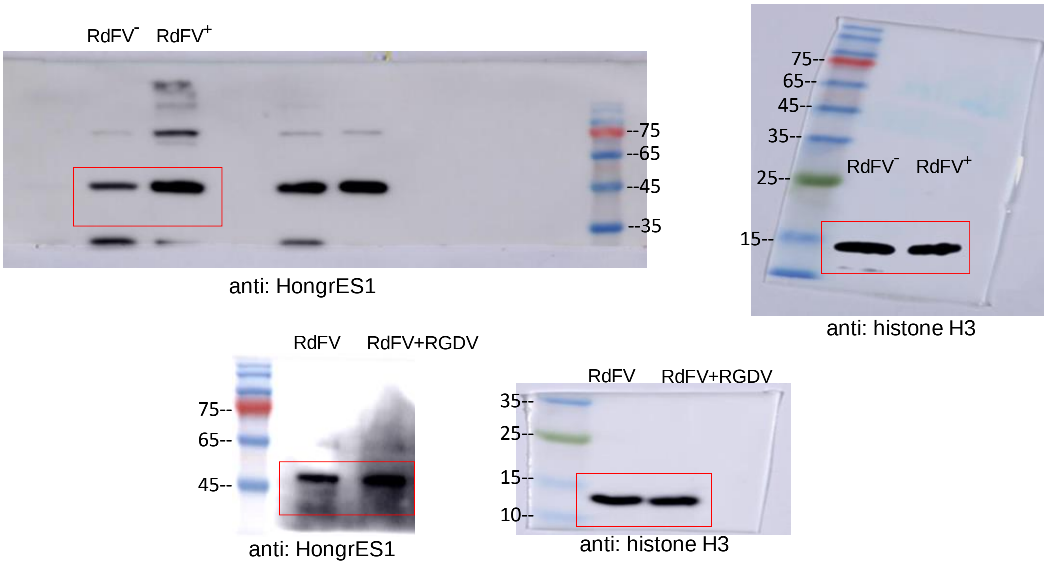

Fig. 4f

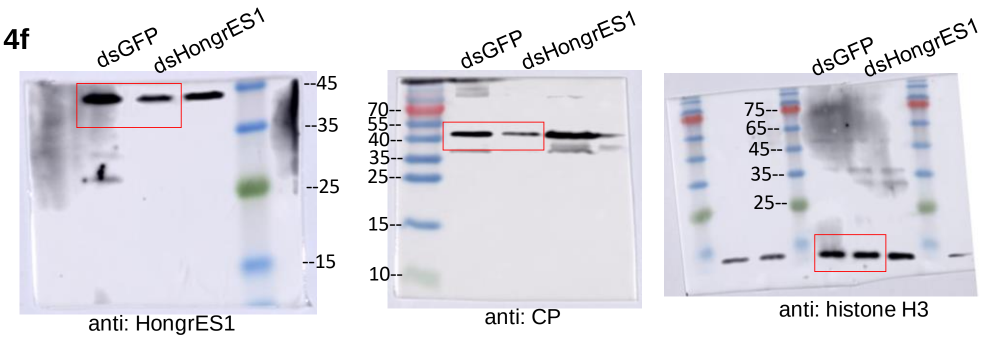

Fig. 4h

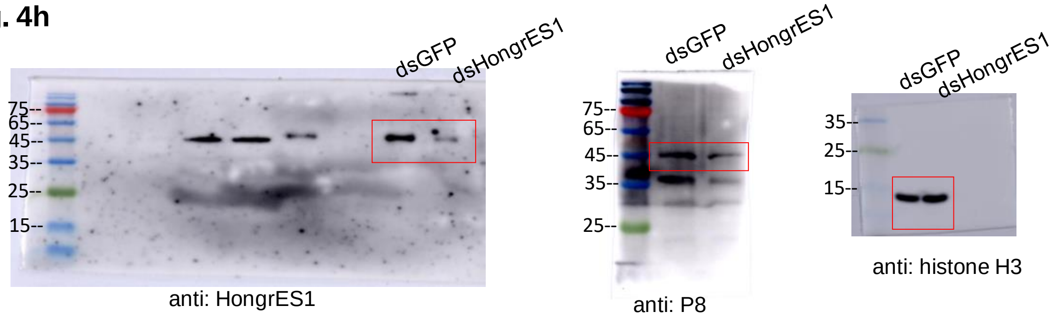

Fig. 5n

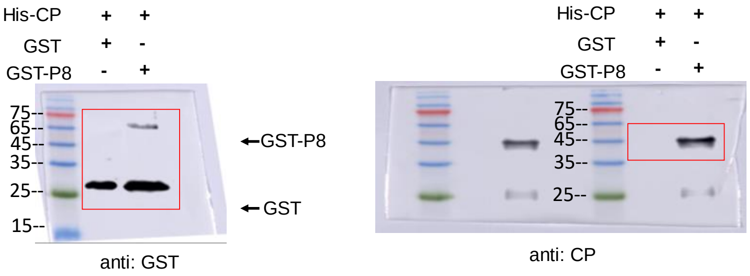

Fig. 6c

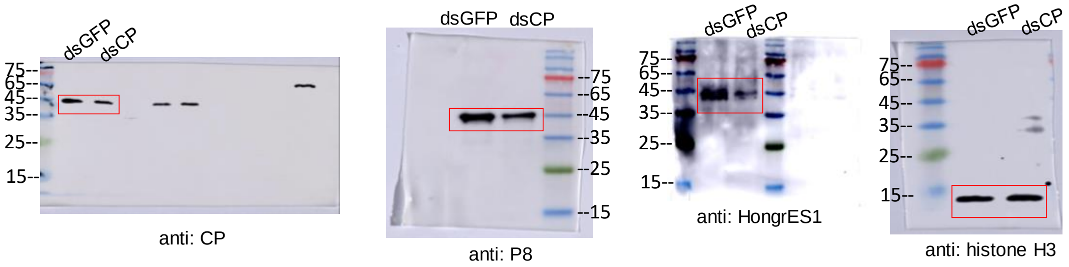

Fig. 6d

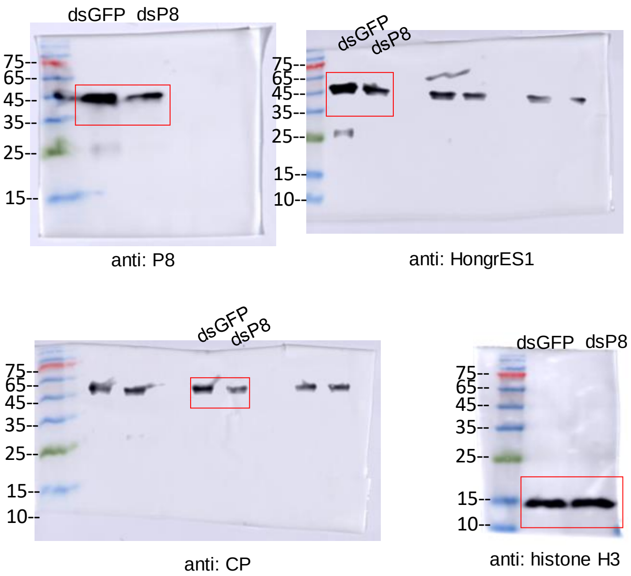

Fig. 7c

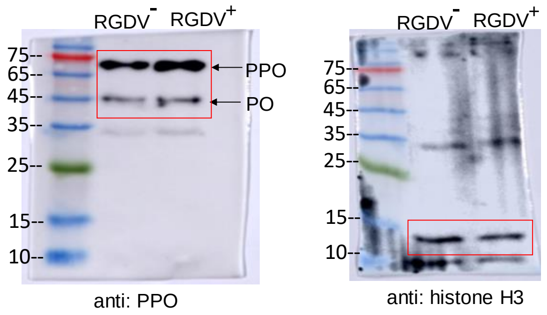

Fig. 7f

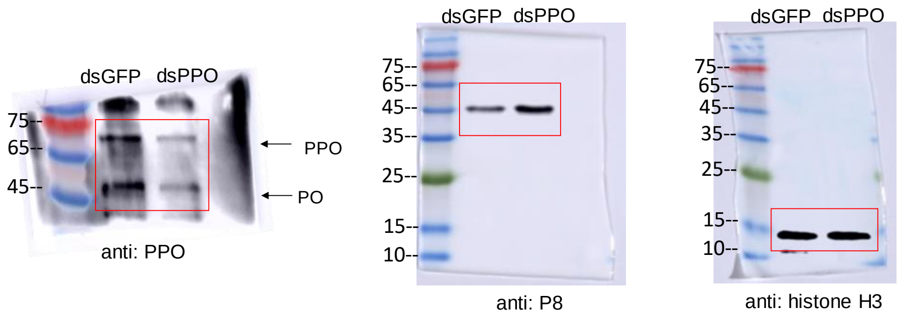

Fig. 7h

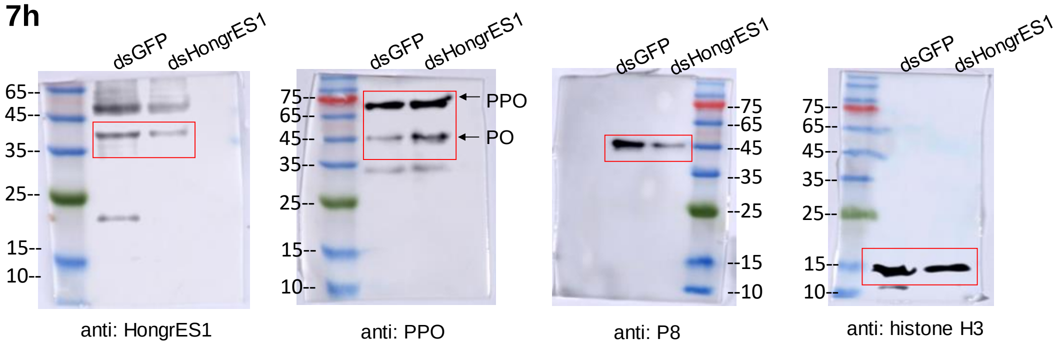

Supplement: Supplementary file 1 — Supplementary information [file 41467_2023_36993_MOESM1_ESM.pdf]
