# Supplementary material for: Arboviruses and symbiotic viruses cooperatively hijack insect sperm-specific proteins for paternal transmission
Source: Nat Commun. 2023 Mar 9;14:1289. doi: 10.1038/s41467-023-36993-0 (PMC9998617; doi:10.1038/s41467-023-36993-0)
Supplement: Supplementary file 3 — Reporting Summary [file 41467_2023_36993_MOESM3_ESM.pdf]

nature portfolio

Corresponding author(s): Taiyun Wei, Qian Chen

Last updated by author(s): Sep 21, 2022

Reporting Summary

Nature Portfolio wishes to improve the reproducibility of the work that we publish. This form provides structure for consistency and transparency in reporting. For further information on Nature Portfolio policies, see our Editorial Policies and the Editorial Policy Checklist.

Statistics

For all statistical analyses, confirm that the following items are present in the figure legend, table legend, main text, or Methods section.

n/a	Confirmed	
☐	☒	The exact sample size (*n*) for each experimental group/condition, given as a discrete number and unit of measurement
☐	☒	A statement on whether measurements were taken from distinct samples or whether the same sample was measured repeatedly
☐	☒	The statistical test(s) used AND whether they are one- or two-sided<br>*Only common tests should be described solely by name; describe more complex techniques in the Methods section.*
☐	☒	A description of all covariates tested
☐	☒	A description of any assumptions or corrections, such as tests of normality and adjustment for multiple comparisons
☐	☒	A full description of the statistical parameters including central tendency (e.g. means) or other basic estimates (e.g. regression coefficient) AND variation (e.g. standard deviation) or associated estimates of uncertainty (e.g. confidence intervals)
☒	☐	For null hypothesis testing, the test statistic (e.g. *F*, *t*, *r*) with confidence intervals, effect sizes, degrees of freedom and *P* value noted<br>*Give P values as exact values whenever suitable.*
☐	☒	For Bayesian analysis, information on the choice of priors and Markov chain Monte Carlo settings
☒	☐	For hierarchical and complex designs, identification of the appropriate level for tests and full reporting of outcomes
☒	☐	Estimates of effect sizes (e.g. Cohen's *d*, Pearson's *r*), indicating how they were calculated

Our web collection on statistics for biologists contains articles on many of the points above.

Software and code

Policy information about availability of computer code

Data collection	High quality RNAs for construction of small RNAs and transcriptomic libraries were sequenced using an Illumina NovaSeq 6000 platform in Novogene Co., Ltd, China.
Data analysis	The Bayesian inference in MrBayes 3.2.6 under the rtREV+F+G4+I model was applied for phylogeny analysis, and Graphpad 6.0 was applied for statistical analyses.

For manuscripts utilizing custom algorithms or software that are central to the research but not yet described in published literature, software must be made available to editors and reviewers. We strongly encourage code deposition in a community repository (e.g. GitHub). See the Nature Portfolio guidelines for submitting code & software for further information.

Data

Policy information about availability of data

All manuscripts must include a data availability statement. This statement should provide the following information, where applicable:

- Accession codes, unique identifiers, or web links for publicly available datasets
- A description of any restrictions on data availability
- For clinical datasets or third party data, please ensure that the statement adheres to our policy

The authors declare that all data supporting the findings of this study are available in the manuscript and its Supplementary Information files are available from the corresponding authors upon request.

Human research participants

Policy information about studies involving human research participants and Sex and Gender in Research.

Reporting on sex and gender	This study did not involve human research.
Population characteristics	This study did not involve human research.
Recruitment	This study did not involve human research.
Ethics oversight	This study did not involve human research.

Note that full information on the approval of the study protocol must also be provided in the manuscript.

Field-specific reporting

Please select the one below that is the best fit for your research. If you are not sure, read the appropriate sections before making your selection.

☒ Life sciences ☐ Behavioural & social sciences ☐ Ecological, evolutionary & environmental sciences

For a reference copy of the document with all sections, see nature.com/documents/nr-reporting-summary-flat.pdf

Life sciences study design

All studies must disclose on these points even when the disclosure is negative.

Sample size	Fifty females mated with 50 male adults to create each mating combination population to determine vertical transmission of RdFV.<br>Approximate 200 leafhoppers were analyzed for the expression levels of HongrES1 in different tissues.<br>Approximate 300-500 2nd-instar or 3rd-instar nymphs of leafhoppers were fed on RGDV-diseased rice plants to acquire virus.<br>Approximate 500 3rd-instar nymphs of leafhoppers were microinjected with a mixture of purified RGDV viruses or were knockdown of genes expression of HongrES1 or CP.
Data exclusions	No data were excluded from the analyses.
Replication	Data of vertical transmission of virus, fitness measurement of leafhoppers, mating experiments, RT-qPCR assays and western blot assays represent three biological replicates.
Randomization	Leafhopper or rice samples were randomly collected for experimental groups.
Blinding	We were blinded to group allocation during data collection and analysis.

Reporting for specific materials, systems and methods

We require information from authors about some types of materials, experimental systems and methods used in many studies. Here, indicate whether each material, system or method listed is relevant to your study. If you are not sure if a list item applies to your research, read the appropriate section before selecting a response.

Materials & experimental systems

n/a	Involved in the study	
☐	☒	Antibodies
☒	☐	Eukaryotic cell lines
☒	☐	Palaeontology and archaeology
☐	☒	Animals and other organisms
☒	☐	Clinical data
☒	☐	Dual use research of concern

Methods

n/a	Involved in the study	
☒	☐	ChIP-seq
☒	☐	Flow cytometry
☒	☐	MRI-based neuroimaging

Antibodies

Antibodies used	Rabbit polyclonal antibodies against RdFV CP, HongrES1, RGDV P8 and PPO were prepared by Genscript Biotech Corporation, Nanjing, China. Mouse monoclonal antibodies against 6×His tag and GST were purchased from Transgene Biotech (HT501; Beijing, China). The actin dyes phalloidin-Alexa Fluor 647 carboxylic acid and 4’,6-diamidino-2-phenylindole (DAPI) were purchased from Thermo Fisher Scientific (A22287, 62248; Waltham, MA, USA). Rabbit polyclonal antibody against Histone H3 was purchased from Abcam (ab1791;

	Cambridge, UK).
Validation	Rabbit polyclonal antibodies against RdFV CP, HongrES1, RGDV P8 and PPO antigens were prepared by Genscript Biotech Corporation, Nanjing, China. The process was approved by the Science Technology Department of Jiangsu Province of China. The validation statements of commercial antibodies are available on the manufacturer's website.

Animals and other research organisms

Policy information about studies involving animals; ARRIVE guidelines recommended for reporting animal research, and Sex and Gender in Research

Laboratory animals	Rice green leafhopper Recilia dorsalis.
Wild animals	The RdFV-positive, RGDV-infected and free leafhopper R. dorsalis adults were originally collected from rice fields in Luoding, Guangdong Province, China.
Reporting on sex	These findings demonstrated vertical transmission of virus in female and male leafhoppers. Female and male leafhoppers were considered and compared in study design.
Field-collected samples	The RdFV-positive and free leafhopper R. dorsalis adults collected from rice fields were maintained in insect-proof greenhouses at 25 ± 3°C on TN-1 rice plants. RGDV-infected leafhopper R. dorsalis adults and rice plants were originally collected from Luoding, Guangdong Province, China. RGDV-infected rice plants were propagated via transmission by RGDV-infected R. dorsalis under greenhouse conditions.
Ethics oversight	No ethical approval or guidance was required because the materials were insects and plant.

Note that full information on the approval of the study protocol must also be provided in the manuscript.
